# Primate-specific stress-induced transcription factor POU2F1Z protects human neuronal cells from stress

**DOI:** 10.1038/s41598-021-98323-y

**Published:** 2021-09-22

**Authors:** Alexander G. Stepchenko, Tatiana N. Portseva, Ivan A. Glukhov, Alina P. Kotnova, Bella M. Lyanova, Sofia G. Georgieva, Elizaveta V. Pankratova

**Affiliations:** 1 Department of Transcription Factors, Engelhardt Institute of Molecular Biology, Russian Academy of Sciences, Moscow, Russia; 2 Center for Precision Genome Editing and Genetic Technologies for Biomedicine, Engelhardt Institute of Molecular Biology, Russian Academy of Sciences, 119991 Moscow, Russia

**Keywords:** Cell biology, Developmental biology, Evolution, Molecular biology

## Abstract

The emergence of new primate-specific genes is an essential factor in human and primate brain development and functioning. POU2F1/Oct-1 is a transcription regulator in higher eukaryotes which is involved in the regulation of development, differentiation, stress response, and other processes. We have demonstrated that the Tigger2 transposon insertion into the POU2F1 gene which occurred in the primate lineage led to the formation of an additional exon (designated the Z-exon). Z-exon-containing primate-specific Oct-1Z transcript includes a short upstream ORF (uORF) located at its 5’-end and the main ORF encoding the Oct-1Z protein isoform (Pou2F1 isoform 3, P14859-3), which differs from other Oct-1 isoforms by its N-terminal peptide. The Oct-1Z-encoding transcript is expressed mainly in human brain cortex. Under normal conditions, the translation of the ORF coding for the Oct-1Z isoform is repressed by uORF. Under various stress conditions, uORF enables a strong increase in the translation of the Oct-1Z-encoding ORF. Increased Oct-1Z expression levels in differentiating human neuroblasts activate genes controlling stress response, neural cell differentiation, brain formation, and organogenesis. We have shown that the Oct-1Z isoform of the POU2F1/Oct-1 transcription factor is an example of a primate-specific genomic element contributing to brain development and cellular stress defense.

## Introduction

The POU2F1/Oct-1/OTF1 transcription factor (further referred to as Oct-1) encoded by the *POU2F1* gene belongs to the family of DNA-binding POU domain-containing transcription regulators of higher eukaryotes. Oct-1 controls the expression of a large number of genes involved in development, differentiation, and stress response^1–4^. The broad range of Oct-1 targets includes house-keeping genes^5–8^ along with the immune system, endocrine system, and nervous system-specific genes^9–12^. Oct-1 is considered to be an important regulator of cancer stem cells^2,13,14^. Oct-1 removal leads to the selective depletion of stem-like populations in multiple human tumor cell lines^2^. Recent studies have shown an important role for high Oct-1 levels and its predictive value in malignant tumor development^15^. Oct-1 is essential for proper embryo implantation and for normal tissue development in mice embryos at the gastrula stage^16^. In humans, Oct-1 expression starts at the preimplantation embryo stage^17^.

Oct-1 participates in the nervous system and brain development in different organisms and is among the transcription regulators, which are actively expressed in the developing nervous system^16–19^. Mouse embryonic stem cells with artificially generated deficiency for Oct-1 do not properly differentiate into neurons^20^. At the later stages of embryogenesis in mice, Oct-1 controls lens and olfactory placode development^21^. Oct-1 plays an important role in the cell-specific and hormonal regulation of GnRH gene transcription in the cell lines, derived from the gonadotropin-releasing hormone (GnRH) neurons of mouse hypothalamus^22^. Oct-1 is necessary and sufficient for radial glia formation preceeding the neural tube closure in Xenopus^23^. High levels of Oct-1 expression in the germinal zones of human fetal brain were observed in the course of human cortex development^24^.

Several Oct-1 isoforms which differ by their N-terminal and C-terminal regions have been described^1,25–30^. In particular, the ubiquitously expressed Oct-1A (POU2F1 transcript variant 3, NM002697, Uniprot P14859-6) isoform and Oct-1L (POU2F1 transcript variant 2, NM_001198783, Uniprot P14859-2) isoform, which is predominantly expressed in B-cells and brain, differ in their N-terminal peptides^1,25–30^. The murine isoform (AY177625, PIAA045298.1) designated Oct-1Z lacks a large C-terminal part, but still can bind the Oct-1 motif and activate basal promoter activity^30^. Some Oct-1 isoforms were predicted but not yet characterized, in particular the isoform encoded by the Oct-1Z transcript (POU2F1 transcript variant 5, NM_001365848.1, Uniprot P14859-3)^4^ (note, it is not related to the mice Oct-1Z isoform). Oct-1 isoforms reveal similarities and, at the same time, significant differences in the arrays of their targets in human cells^3,4^, suggesting that further study of novel Oct-1isoforms is important for the understanding of the *POU2F1* gene functioning.

The important role of Oct-1 in stress response has been demonstrated^3,31^. A number of cellular stress-associated genes exhibit altered expression in Oct-1-deficient mouse fibroblasts^31^. Oct-1-deficient fibroblasts were hypersensitive to a number of stress agents including γ-rays, doxorubicin, and hydrogen peroxide. Oct-1-deficient cells responded abnormally to cellular stress, while many genes associated with oxidative and metabolic stress were dysregulated in Oct-1-deficient fibroblasts following radiation exposure^31^. The Oct-1 DNA-binding domain is phosphorylated under stress conditions, which results in the altered affinity of the domain to its binding sites^21^.

Most cellular stresses such as Endoplasmic Reticulum (ER) stress, hypoxia, viral infection result in the accumulation of unfolded proteins in the lumen of Endoplasmic Reticulum (ER) causing ER stress^32^. ER stress triggers the activation of the unfolded protein response (UPR), which leads to a global decrease in translation initiation levels. To survive under stress conditions, cells utilize alternative mechanisms of translation initiation for a number of proteins involved in stress response such as ATF4, XIAP, or p53^33^. Protein synthesis in cell stress response is coordinated by posttranscriptional mechanisms. Some of them act to increase the synthesis of the key modulators of damage response. Some transcripts which are translated during stress contain an internal ribosome entry site (IRES)^34,35^. Another mechanism of alternative translation initiation relies upon the presence of a short ORF (uORF) upstream of the main protein-coding ORF in the transcript. The mechanism of translation reinitiation employing uORF is conserved from yeasts to mammals^36,37^. Upstream ORFs (uORFs) are the transcription control elements found predominantly in the transcripts of the key regulatory genes, for example ATF4, C/EBPb, and GADD34^37–40^. Under stress conditions, the uORF enables translation initiation from the internal ATG. While the mechanism of this typeof translation is not completely understood seemingly depending on the particular mRNA structure, this mechanism is widely exploited to enable protein translation during stress^32,33,40^.

In the present work, we investigated the function of the Oct-1Z isoform (P14859-3) and the structure of the corresponding transcript. We have demonstrated that the POU2F1 gene contains a primate-specific exon (further referred to as Z-exon) which originates from the Tigger2 transposon insertion along the primate linage. The regulatory mechanism of the Z-exon containing-transcript translation is different from that of other Oct-1 transcripts. Z-exon carries a stop-codon and an alternative translation start site downstream from it. This results in a short uORF located upstream of the main protein-coding sequence. As a result, under normal conditions, the translation level of the Oct-1Z isoform is low. However, under various stress conditions, it strongly increases while the translation of other Oct-1 transcripts drops down. Among human tissues, cerebral cortex has the highest amounts of Oct-1Z transcripts. To study the role of Oct-1Z in stress response in neural cells, human neuroblastoma cell line (IMR-32) was chosen. When in the differentiated state, it was shown to mimic the large projections of human cerebral cortex^41^ and was previously used to study different aspects of neural cells functioning including stress response^42^. In differentiating human IMR-32 neuroblastoma cells, Oct-1Z overexpression activates the signaling pathways that regulate stress response, neuronal differentiation, and brain development. We conclude that the increased levels of Oct-1Z isoform expression in human neural cells enhance their stress resistance.

## Results

### The Tigger2 transposon insertion into the *POU2F1* gene of primates gave rise to a novel transcript (Oct-1Z) which contains the Tigger2-derived Z-exon and a short uORF resulting from its presence

**Figure 1:**
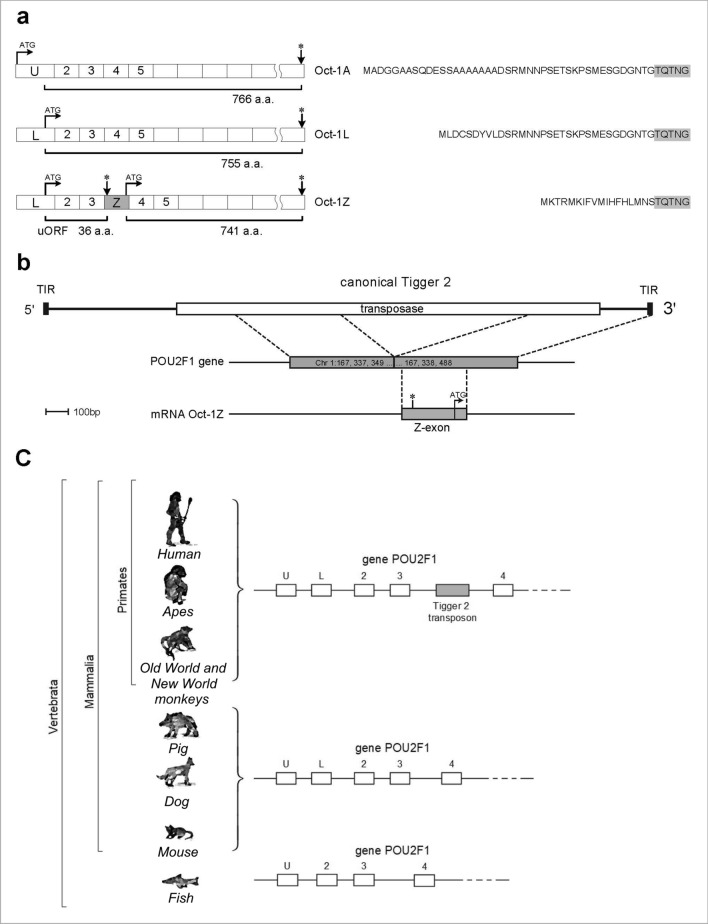
Schematic representation of Oct-1 transcripts. (**a**) Structure of the Oct-1L and Oct-1Z transcripts with different internal exons and the amino acid sequences of the differing N-terminal domains of the Oct-1L and Oct-1Z isoforms. Translation start sites are indicated with black arrows. Stop codons are indicated with asterisks. (**b**) Formation of the Oct-1Z transcript by alternative splicing which results in the partial Tigger2 transposon inclusion into the mRNA. (**c**) Schematic representation of the 5'-end of the POU2F1 locus in vertebrates and the emergence of the Tigger 2 transposon in the POU2F1 gene in primates.

Aiming to characterize the Oct-1Z isoform we first studied the structure of the corresponding transcript. Previously, two Oct-1transcripts starting from the alternative promoters have been described (Fig. 1a). The transcript Oct-1A encodes the ubiquitously expressed Oct-1A isoform, while the transcript Oct-1L encodes the tissue-specific isoform L^3^. These isoforms differ by their short N-terminal peptides (Fig. 1a). The Oct-1Z transcript contains an additional exon (Fig. 1a), further referred to as Z-exon, and may start from the two alternative promoters (MT294127 and AK091438.1). Notwithstanding the resulting RNAs differing by their 5’-ends, identical proteins, which are both the Oct-1Z isoform (UniProt P14859-3), are translated from them. The presence of the Z-exon leads to the changes in the ORF composition of the transcript. Z-exon contains a stop codon, which results in the formation of a short upstream open reading frame (uORF). The Z-exon also contains an ATG codon downstream of the stop codon, which, in its turn, gives start to a long ORF, which is in the same frame as the other Oct-1 protein isoforms, but contains a unique short N-terminal sequence (Fig. 1a).

We have found that Oct-1Z isoform emerged as a result of the Tigger2 transposon fragment insertion into the *POU2F1* intron with the subsequent formation of the new primate-specific Z-exon (Fig. 1b). Database survey revealed that Z-exon exists only in the *POU2F1* gene of humans and other primates including the Old World and New World monkeys (Fig. 1c). In line with that Oct-1Z transcript was detected only in human and primate tissues.

The Tigger2 DNA transposon belongs to the DNA/TcMar-Tigger family. It possesses 24 bp inverted terminal repeats and a single open reading frame which encodes the transposase (Fig. 1b). Tigger2 insertion into the *POU2F1* gene has the following genomic coordinates: hg38:chr1:167,337,938–167,338,488. This is a defective Tigger2 copy bearing two large deletions, namely, a 974 bp deletion within the proximal end and a 775 bp deletion in the central region (Fig. 1b). The original structure of the fusion gene is conserved in all anthropoid lineages.

### Compared to the ubiquitously expressed Oct-1A transcript the new primate-specific Oct-1Z transcript has its maximum expression level in human brain, in particular, in the cortex

We have previously studied the expression levels of the ubiquitous Oct-1A transcript (NM002697, UniProt P14859-6) and have demonstrated them to be relatively high in different human tissues and particularly high in brain^3^. To study the Oct-1Z transcription, mRNA samples from different human tissues were used. As we have shown previously, the main Oct-1 transcript, Oct-1A, is ubiquitously expressed in human tissues^3^. Here, we have compared Oct-1Z and Oct-1A transcription levels. Although it is not possible to use the ubiquitously expressed Oct-1A transcript for normalization because its expression varies between tissues, higher transcription level of Oct-1Z relative to the general Oct-1A level may suggest functional importance of Oct-1Z in the studied tissue.

**Figure 2:**
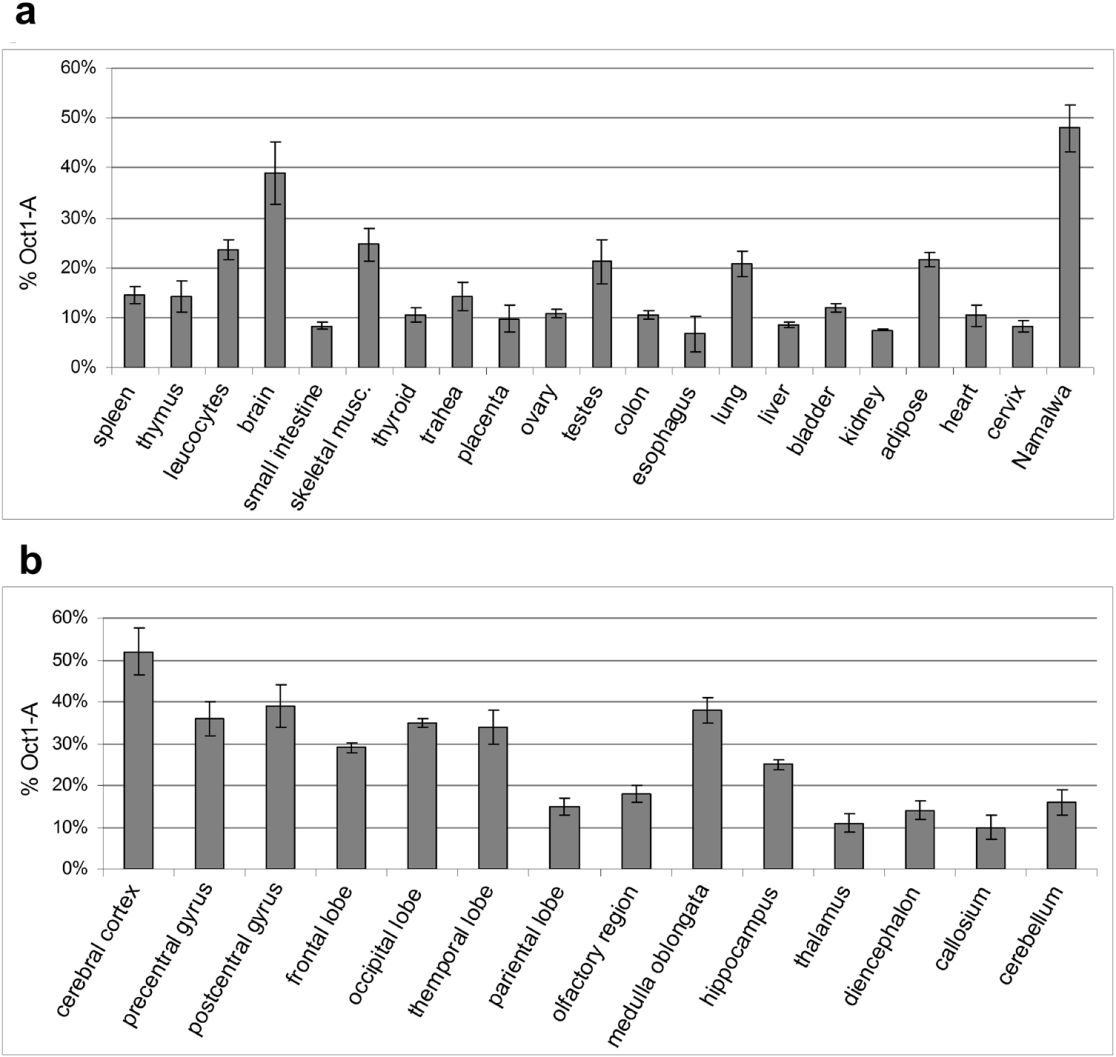
Oct-1 isoforms expression at the mRNA level in human cells measured by Real-Time PCR. (**a**) Relative Oct-1Z isoform mRNA levels in human tissues. (**b**) Relative Oct-1Z isoform mRNA levels in human brain regions. Oct-1Z mRNA level is normalized to the ubiquitous Oct-1A isoform mRNA level in each tissue. Oct-1Z and Oct-1A isoform mRNA levels were calculated relative to the GUS mRNA. Oct-1Z expression level relative to Oct-1A was calculated using the ∆∆Ct method. Mean ± S.E.M. for three independent experiments are provided. RNA samples were isolated from the human tissues which may contain non-neuronal human cells.

In most tissues, Oct-1Z was detected at the levels constituting about 10–15% of the Oct-1A isoform expression level (Fig. 2a). However, Oct-1Z transcript levels in brain were significantly higher (about 40% of the Oct-1A level). The maximum Oct-1Z expression relative to Oct-1A was observed in brain cortex (Fig. 2b).

In summary, Oct-1Z transcript has arisen in the primate lineage, with high levels of its expression relative to Oct-1A expression being detected in brain, in particular, in cortex, which may serve as an indirect evidence for the functional importance of the protein encoded by this transcript in brain.

### Translation of the ORF coding for the Oct-1Z protein is inhibited by uORF

**Figure 3:**
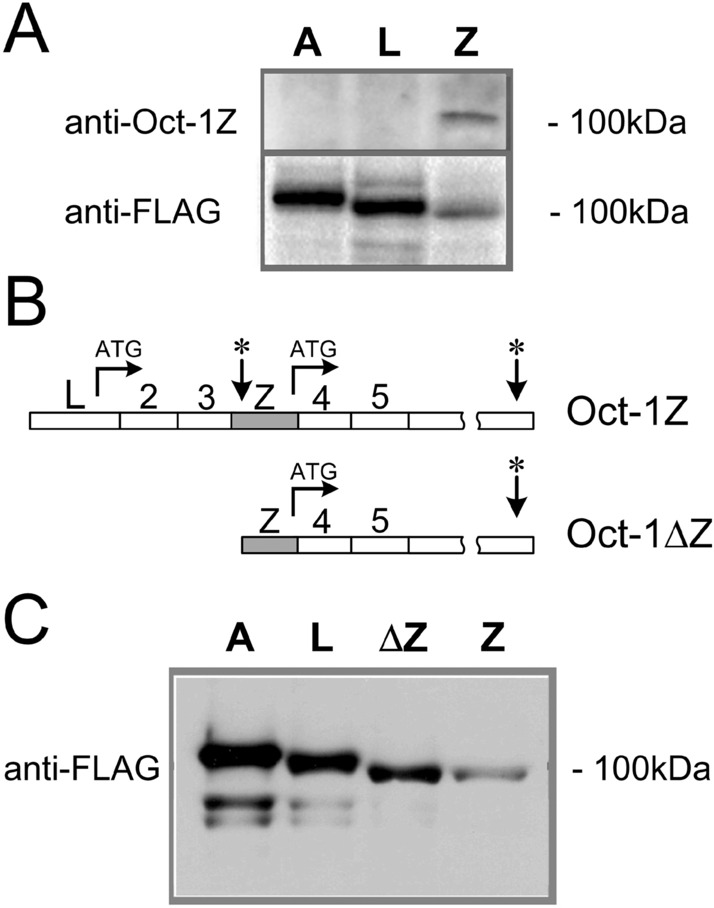
Western blot analysis of in vitro transcribed and translated Oct-1 isoforms. (**a**) In vitro transcription and translation of Oct-1A (line A), Oct-1L (line L), and Oct-1Z (line Z) isoforms. Anti-Oct-1Z antibodies specifically recognize the Oct-1Z, but not the Oct-1A and Oct-1L isoforms. (**b**) Schematic representation of the Oct-1Z and Oct-1ΔZ ORFs cloned into the pcDNA3.1 vector. In Oct-1∆Z, 285 bp starting from the ATG codon of the L-exon were deleted. (**c**) The 5’-end uORF deletion enhances the translation of the Oct-1Z isoform. Anti-FLAG antibodies were used to detect Oct-1A, Oct-1L, Oct-1ΔZ, and Oct-1Z. 1 ug of the pcDNA3.1-Oct-1-constructs were taken into the coupled transcription–translation reactions. Whole-size Western blots images are provided in Supplementary Figure S1.

The polyclonal antibodies against the N-terminal peptide specific to Oct-1Z were raised in rabbits. To test the antibodies, Oct-1Z, Oct-1A, and Oct-1L isoforms conjugated with the C-terminal FLAG epitope were produced in the in vitro transcription-translation system. The fragment of the Oct1-Z cDNA containing both uORF and the Oct-1Z-encoding ORF was cloned into pcDNA3.1. The Oct-1A and Oct-1L ORFs were also cloned into pcDNA3.1. The antibodies against Oct-1Z specifically recognized the Oct-1Z protein (about 100 kD), but not the Oct-1L or Oct-1A isoforms produced in the in vitro transcription-translation system (Fig. 3a), while the anti-FLAG antibodies recognized all three isoforms. As expected, the molecular weight of the Oct-1Z protein was slightly lower than that of Oct-1L or Oct-1A.

Significantly, Oct-1Z mRNA translation level was much lower than that of the Oct-1A or Oct-1L transcripts (Fig. 3a). We hypothesized that the uORF present in the Oct-1Z transcript may inhibit the translation of the Oct-1Z isoform. Indeed, the deletion of the uORFregion (285 nucleotides starting from the ATG codon) (Oct-1ΔZ) (Fig. 3b) led to a strong increase in the levels of Oct-1Z produced in the in vitro transcription–translation system (Fig. 3c, Supplementary Fig. S1), making it comparable with that of Oct-1L.

### Oct-1Z isoform level is increased under stress conditions

It has been demonstrated that the presence of an uORF in mRNA may be a part of the mechanism underlying the stress resistance of such mRNA translation^32,33,40^. Under normal conditions, the upstream uORF may strongly repress the translation of the main downstream ORF. However, under stress conditions, the translation mechanism for these mRNAs is changed, which leads to a strong increase in the downstream ORF translation.

**Figure 4:**
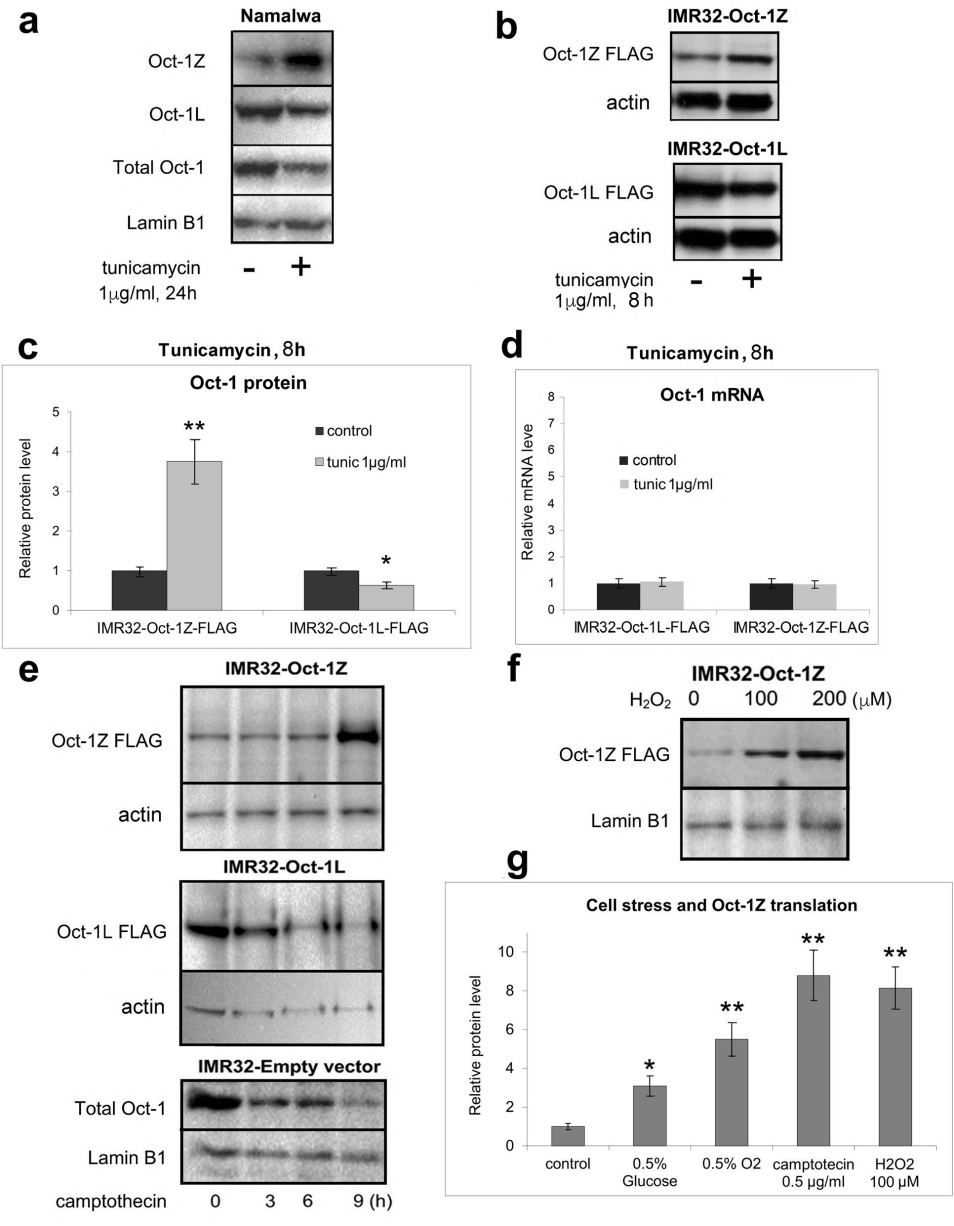
The effects of cellular stress on the translation of Oct-1 isoforms in human cells. (**a**) Western blot demonstrating the expression of the endogenous Oct-1 isoforms in the Namalwa cell line under normal and tunicamycin-induced endoplasmic reticulum (ER) stress conditions. Western blots were probed with the antibodies against Oct-1Z, Oct-1L, and total Oct-1, respectively. Lamin B was used as a loading control. Quantitative assessment results are provided in Supplementary Fig. 2. (**b**) Western blot demonstrating the expression of the recombinant Oct-1Z and Oct-1L isoforms in the stably transduced IMR32 cell line under normal and ER stress conditions. (**c**,**d**) Tunicamycin-induced ER stress causes the induction of the synthesis of the Oct-1Z protein and a decrease in the synthesis of the Oct-1L protein (**c**), but not of the corresponding mRNA. GUSB gene was used to normalize qPCR data. (**e**) Camptothecin-induced stress triggers the expression of the Oct-1Z protein in the IMR32-Oct1Z cells, but not of the Oct-1L or total Oct-1 protein in the IMR32-Oct-1L or IMR32-Em.V. cells. (**f**) Hydrogen peroxide-induced stress triggers Oct-1Z protein expression. (**g**) Various types of stress (as indicated) cause the induction of Oct-1Z protein synthesis in the IMR32-Oct1Z cell line. (**c**,**d**,**g**). Mean ± S.E.M. for three independent experiments are provided. *t*-tests were performed to determine whether there is a significant difference between the control and treated cell means (*P < 0.05 and **P < 0.01). Lamin B or b-actin was used as a loading control. Antibodies against FLAG were used to detect the Flag-tagged Oct-1 overexpressed in the IMR32 cells in the Western blots in (**b**–**g**). Full-size blots are presented in Supplementary Fig. 2.

We have tested if stress has an impact on the level of Oct-1Z in the cells. The Namalwa Burkitt’s lymphoma cell culture was used in the experiment, since relatively high levels of Oct-1Z transcription were observed in these cells (Fig. 2a). The Namalwa cells were treated with tunicamycin which induces ER (endoplasmic reticulum) stress and the level of the endogenous Oct-1Z isoform was compared between the control and tunicamycin-treated cells. The level of Oct-1Z isoform significantly increased in response to stress (Fig. 4a). On the opposite, the level of the Oct-1L isoform decreased after tunicamycin treatment (Fig. 4a).

We have further hypothesized that the novel primate-specific Oct-1Z isoform, which is strongly expressed in brain cells, in particular, in the cortex (Fig. 2b), may play a role in stress response in brain cells. To study the effects of stress on Oct-1 translation, human neuroblastoma IMR-32 cells were used. Differentiated IMR-32 mimic the large projections of human cerebral cortex^41,42^. In the course of differentiation, cells change their expression patterns and their phenotype.

To reveal the effects of stress on Oct-1Z translation, neuroblastoma IMR32 cells were stably transformed with either an empty vector (IMR32-Em.V.), or a lentivirus containing either the Oct-1Z cDNA region encompassing uORF and the Oct-1Z-encoding ORF conjugated with the C-terminal FLAG epitope (IMR32-Oct-1Z), or the ORF encoding Oct-1L conjugated with the C-terminal FLAG epitope (IMR32-Oct-1L) as a control. These two cDNA differ only by the presence of Z-exon (Fig. 1). Cells were differentiated in the complete MEM medium containing 2.5 µM BrdU for 16 days according to the protocol described in^43,44^ and treated with different stress agents. The BrdU protocol is optimal for IMR32 differentiation, as it results in the most clearly differentiated phenotype compared to the differentiation in the presence of other agents (e.g. retinoic acid) (Supplementary Figure S3).

The differentiated IMR32 cells were treated with tunicamycin for 8 h. The results of the Western-blot analysis (Fig. 4b) demonstrated that under the tunicamycin-induced ER stress, the amount of Oct-1Z protein increased by about 4 times in the cells, with a simultaneous decrease in the amount of the Oct-1L isoform. The data on the changes in the Oct-1L and Oct-1Z levels obtained in several independent experiments are presented in (Fig. 4c). Noteworthy, the levels of Oct-1L and Oct-1Z mRNAs showed no changes after tunicamycin treatment indicating that the observed altered levels of the Oct-1Z and Oct-1L proteins result from the changes in mRNA translation (Fig. 4d).

An increase in the Oct-1Z protein level was also observed under other stress conditions including genotoxic stress (growing cells in the medium containing 0.5 ug of camptothecin, 9 h) (Fig. 4e), oxidative stress (growing cells in the presence of 100 or 200 µM H_2_O_2_, 24 h) (Fig. 4f), hypoxia (0.5% O_2_, 24 h), and low glucose content (growing cells in the DMEM medium containing 0.5% glucose, 24 h) (Fig. 4g, Supplementary Figure S2),). Hence, the synthesis of the exogenous Oct-1Z-FLAG protein increases under all the tested types of cellular stress.

In summary, different types of stress cause strong activation of Oct-1Z mRNA translation. On the contrary, the Oct-1L transcript, which does not contain the Z-exon, is efficiently translated under normal conditions, but its translation decreases in the cells subjected to stress. Thus, the presence of the primate-specific Z-exon in the transcript leads to the formation of the uORF which makes it possible to switch on an alternative translation mechanism. The general principle of the uORF-mediated stress resistance is based on the strong repression of downstream translation under normal conditions, and its activation under stress conditions^40^.

### Oct-1Z activates stress protection response and protects cells from stress

The data presented above indicate that the synthesis of the Oct-1Z protein increases considerably under cell stress conditions. To confirm the role of the Oct-1Z isoform in the development of cellular response to stress, the pGL4-Firefly Luciferase Reporter Vectors with Cellular Stress Response Elements (Promega) system was utilized. The vector used was pGL4*.*42 encompassing the luciferase reporter gene under the control of the hypoxia response element (HRE)-containing promoter which is induced during cell response to oxygen deficiency. The other vector, pGL4.39, contains the endoplasmic reticulum stress response element (ATF6 Activating Transcription Factor 6 response element) which is activated during the protective response to ER stress.

**Figure 5:**
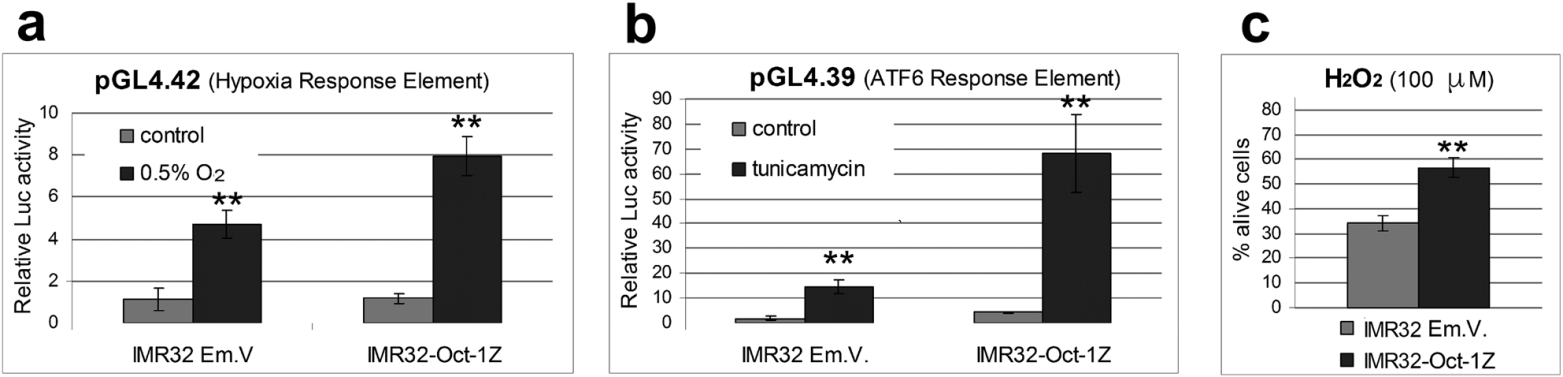
Overexpression of the Oct-1Z isoform in the IMR32 cells activates cellular response to ER-stress, hypoxia, and oxidative stress. (**a**) Oct-1Z stimulates the Hypoxia Response Element-driven luciferase expression in IMR32-Oct1Z under hypoxia. (**b**) Oct-1Z stimulates the ATF6 Response Element-driven luciferase expression in IMR32-Oct-1Z cells under ER-stress. Firefly luciferase luminescence was normalized to Renilla luciferase luminescence. (**c**) Oct-1Z overexpression increases the survival rate of IMR32-Oct1Z cells under oxidative stress compared to the control cells transformed with the empty vector (IMR32 Em.V.). Cell viability was measured using the CellTiter 96 AQ One Solution Assay. Error bars indicate S.E.M. for six replicates. *t*-tests have been performed to compare the means (**P < 0.01).

The IMR32-Oct-1Z or IMR32-Em.V. were differentiated and transfected with the pGL4*.*42 or pGL4.39 reporter vectors and the pRL-TK vector. Dual luciferase assay revealed that Oct-1Z isoform overexpression in hypoxia led to a marked increase in the transcription driven by the hypoxia response element (Fig. 5a). In tunicamycin-induced ER stress, the overexpression of this isoform caused a considerable increase in the transcription controlled by the ATP6 response element compared to the cells transfected with the empty vector (Fig. 5b). These experiments confirm that Oct-1Z activates stress signaling pathways responsible for stress protection.

We have also demonstrated that Oct-1Z increases cell survivability under stress conditions. The IMR32-Oct-1Z and control IMR32-Em.V. cells were subjected to oxidative stress (Fig. 5c). We observed that the viability of IMR32-Oct-1Z was about two times higher compared to the control cells transformed with the empty vector, thus confirming the involvement of Oct-1Z in stress response.

### Oct-1Z activates the genes responsible for neural cells protection from stress

To further study the role of Oct-1Z in gene expression regulation, RNA-Seq was performed. Human IMR32-Oct-1Z and IMR32-Em.V. neuroblastoma cell lines were differentiated for 16 days in the complete MEM medium containing 2.5 µM BrdU. mRNA was then isolated from the cells, and the transcriptome analysis was carried out by RNA-Seq. Endogenous Oct-1Z levels in the IMR32 cells were very low and undetectable by antibodies. Oct-1Z-FLAG levels in the transformed cells are shown on Fig. 4. The IMR32-Em.V. cells were used as a control. Three independent biological replicates were made for the IMR32-Oct-1Z and for the IMR32-Em.V. cells.

RNA-Seq data (GEO GSE153980) confirmed that approximately 8% of the transcripts showed differential expression between the IMR32-Empty Vector and IMR32-Oct-1Z differentiating neuroblastoma cells, with FDR < 0.01 (p-value corrected for multiple testing). The transcription levels of 771 DEGs increased, while the transcription levels of 436 DEGs decreased. The analysis of DEGs demonstrated that the increase in the Oct-1Z levels exerted several major effects on differentiating human neural cells. It enhanced cellular stress response, regulated neuronal differentiation, strengthened organogenesis, and repressed cell cycle progression.

**Figure 6:**
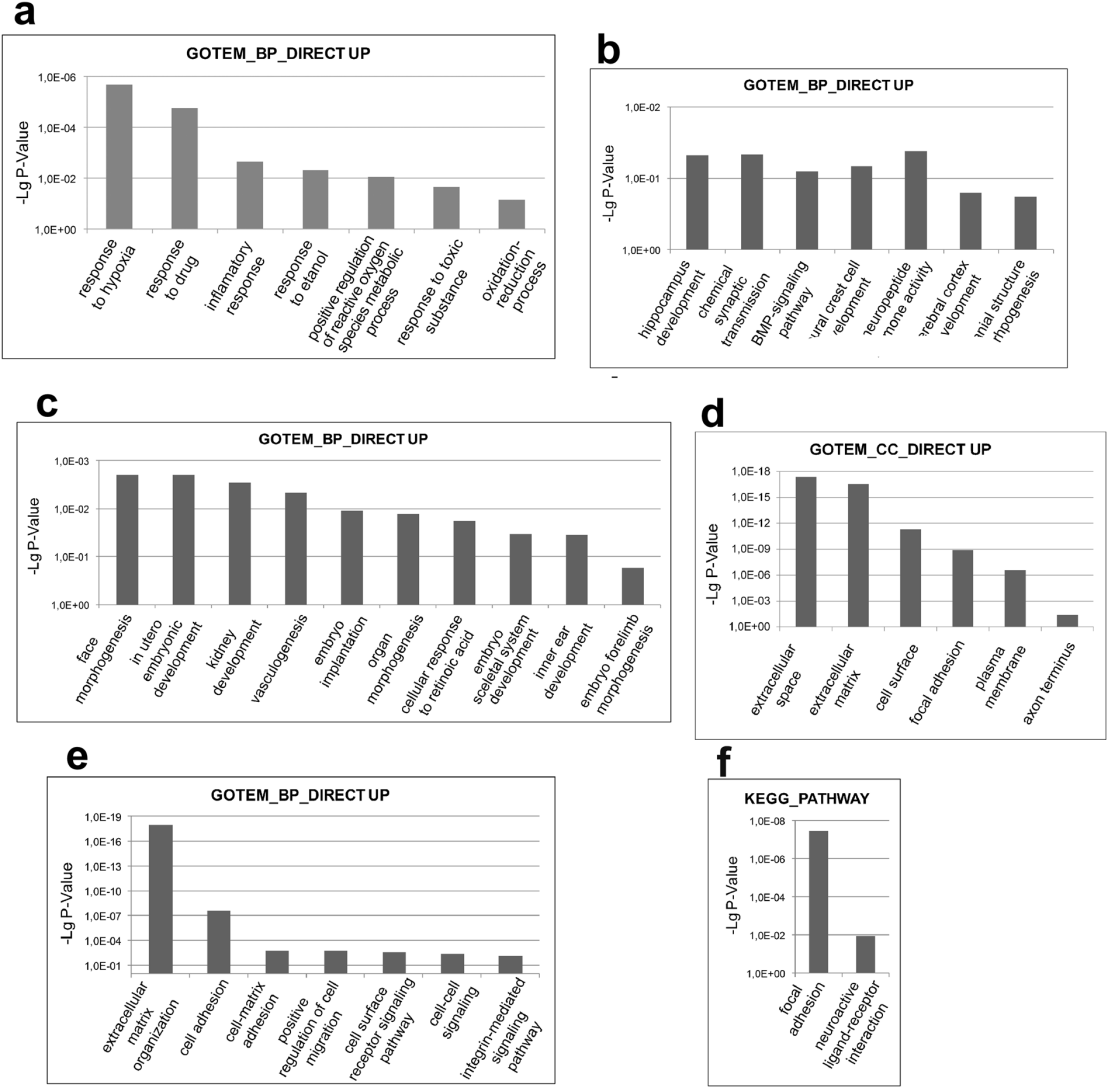
Functional enrichment analysis of differentially expressed genes for the Oct-1Z isoform. The most relevant Gene Ontology (GO) GOTERM BP FAT (biological process) (**a**) related to stress response, (**b**) related to brain development, (**c**) related to organogenesis, (**d**) GOTERM CC FAT (cell components), (**e**) GOTERM BP FAT (biological process) related to cell–matrix and cell–cell interaction, (**f**) KEGG Pathway. Enrichment items of up-regulated DEGs (www.kegg.jp/kegg/kegg1.html).

Oct-1Z regulates those cellular processes which form basis of stress resistance in neural cells. DEG analysis (Functional Annotation DAVID Bioinformatic Resourses 6.8) revealed that the up-regulated genes activate cellular stress response through such cellular processes (GOTEM_BP_DIRECT) as the response to hypoxia, drugs, and ethanol and the positive regulation of reactive oxygen species and toxic substances metabolism, oxidation–reduction processes, and inflammation response (Fig. 6a). Response to lipopolysaccharides, defense response to viruses, response to wounding, and response to oxygen, nutrient, and temperature levels and osmotic stress were also among the up-regulated processes.

The increase in the Oct-1Z levels in differentiating neural cells leads to an increase in the expression of a number of important genes engaged in cellular stress response (Table S1). Among these are the genes which regulate hypoxia and oxidative stress response (CYP1A1, EGLN3, EPAS1, MMP2, PRKCB, AJUBA, FOXO1, LOXL2, LOXL4, MICAL2, and MOXD1) and genes which regulate the response to toxic substances (HTR1D, DHRS2, BMP2, EPHX1, INMT, MAOB, and TLR2).

The increase in the Oct-1 expression level also led to the activation of 34 genes involved in the inflammatory response including such genes as NFKB2, F2RL1, PTX3, TLR2, and others.

### Oct-1Z activates genes involved in the processes associated with brain development and differentiation of neural cells

Oct-1Z is involved in the processes associated with brain development and neuronal differentiation. It up-regulates the genes which participate in the biological processes of hippocampus development, neural crest cell development, brain development, frontal structure morphogenesis, neuropeptide hormone activity, serotonin receptor signaling pathway, chemical synaptic transmission (GOTEM_BP_DIRECT) (Fig. 6), and neuroactive ligand-receptor interactions (KEGG_PATHWAY).

The increase in the Oct-1Z level in differentiating neural cells leads to an increase in the expression of such important genes engaged in the forebrain development as RELN, TBX3, FABP7, MECOM, BCAN, DLX1, NOG, NR2E1, COL3A1, NEFL, NTRK2, NCOA1, and PTPRC (Table S2). These genes regulate forebrain formation by participating in the exceptionally diverse array of processes.

The increase in the Oct-1Z level leads to an increase in the expression of such important genes engaged in the chemical synaptic transmission and neuroactive ligand-receptor interactions as HTR1D, HTR2B, HTR2C, HTR4, ADRB2, CHRNA6, CHRNA9, GABRE, GABRP, GRIN2A, NPY1R, PRLHR, CARTPT, CACNA1E, CRH, PMP22, PDE7B, KCNC4, PENK,PLP1, SLC12A4, SLC6A4, SSTR1, SST, SYPL1, and TACR1 (Table S3).

Down-regulated genes are associated with such processes as Cell division, G1/S transition, G2/M transition (GOTEM_BP_DIRECT); cell cycle, and DNA replication (KEGG_PATHWAY). It is possible that cell cycle suppression is not as much associated with the direct effects of Oct-1Z on these genes, as with its effects on differentiation.

Oct-1Z activities are not limited to neurospecific processes exclusively, the Oct-1Z isoform being directly involved in the organogenesis in primates. The effects of Oct-1Z on organogenesis are associated with the up-regulation of genes which are involved in the following biological processes: embryo implantation, cellular response to retinoic acids, face morphogenesis, and others (GOTEM_BP_DIRECT) (Fig. 6c).

### Oct-1Z controls the regulation of the neural cell–cell and cell–matrix interactions

The rise in the Oct-1Z expression levels has a notable impact on the processes related to the interactions between differentiating neural cells and between the cells and the extracellular matrix, cell adhesion, and extracellular matrix rearrangement. The up-regulated genes include 126 genes encoding plasma membrane proteins, and 52 genes encoding the proteins of the extracellular matrix (GOTEM_CC_DIRECT) (Fig. 6d).

DEGs analysis for the up-regulated genes revealed that according to the Functional Annotation DAVID Bioinformatic Resourse 6.8 the changes occur in the following cellular processes (GOTEM_BP_DIRECT) (Fig. 6e): extracellular matrix organization and disassembly (HTRA1, TIMP1, ACAN, BCAN, CTSS, DCN, ENG, FN1, GSN, KLK4, MMP1, MMP2, MMP10, and MMP12), cell matrix adhesion (CD96, COL3A1, ECM2, FERMT2, and FBLN5), integrins (ITGA11, ITGA2, ITGA3, ITGA6, ITGA8, ITGB3, ITGB5, and NPNT), genes involved in collagen catabolic processes, positive regulation of cell migration, cell surface receptor signaling pathway, cell-substrate adhesion, and (KEGG_PATHWAY) (Fig. 6f): focal adhesion. Therefore, Oct-1Z regulates cell spreading, focal adhesion dynamics, and differentiating neural cells migration.

## Discussion

The extremely complex brain structure in primates and humans is associated with the emergence of new genes. We demonstrate here that the new primate-specific Oct-1Z transcript has emerged as a result of the insertion of the deficient copy of the Tigger2 mobile element into the *POU2F1* gene.

The insertion of the mobile element into the *POU2F1* gene has led to the formation of the additional Z-exon containing a stop codon and a downstream ATG codon. This insertion was found in humans, and the described gene structure can be observed only in the anthropoid lineages, while the alternative splicing of Oct-1Z occurs at its maximum level in the human brain cortex cells. The increase in the Oct-1Z expression levels in differentiating human neuroblasts activates the genes controlling stress response, neural cell differentiation, and brain formation.

The important role of Oct-1 in the regulation of stress response in mammalian cells has been described previously. Here, we have demonstrated that in order to retain its expression level during stress, human Oct-1 uses the alternative Oct-1Z transcript, which contains the novel Z-exon. The short upstream open reading frame (uORF), which has formed as a result of Z-exon insertion represses Oct-1Z mRNA translation under normal conditions, but strongly activates it during stress. Wide occurrence of uORFs in mammalian transcriptomes suggests a comprehensive role of uORF-regulated translation in stress and normal physiology. The physiological importance of uORF-mediated translational control in inflammation, as well as during neuronal differentiation has been demonstrated^38,45^.

Interestingly, a group of genes involved in the inflammation response was identified among the genes up-regulated by Oct-1Z. The NFKB group of genes, which expression and important role in neuronal development has been demonstrated previously, was among them^46^. The TLR2 gene plays a fundamental role in pathogen recognition and innate immunity activation. It was also demonstrated that TLR2 is expressed in CNS neurons, and its levels increase in the Parkinson disease brain. The activation of TLR2 in neural cells induces inflammatory response including the secretion of inflammatory cytokines^47^. The F2RL1/PAR2 receptor has been shown to play an important role in the inflammatory response, as well as in the innate and adaptive immunity^48^.

The obtained results demonstrate that primate-specific Oct-1Z is induced by different types of stress, and up-regulates a significant number of stress protection mechanisms in neural cells. For example, the EGLN3 gene encodes the cellular oxygen sensor which catalyzes the post-translational modification of HIF-alpha proteins under normoxic conditions. EPAS1 encodes the transcription factor which is involved in oxygen-regulated gene activation and is activated itself as cellular oxygen levels decrease. The MMP2 protein contributes to oxidative stress by regulating the activity of GSK3beta, while PRKCB is involved in the apoptosis following oxidative damage. The AJUBA is a hypoxic regulator enabling efficient degradation of HIF1A. The FOXO1 transcription factor regulates metabolic homeostasis in response to oxidative stress.

The DHRS2 gene, which encodes a member of the short-chain dehydrogenases/reductases (SDR) family, the enzymes which metabolize many different compounds, is among the genes which regulate the response to toxic substances. There is also the EPHX1 gene, which encodes the epoxide hydrolase which plays a role in both epoxide activation and detoxification. MAOB encodes the enzyme which is located in the outer mitochondrial membrane and which catalyzes the oxidative deamination of biogenic and xenobiotic amines and plays an important role in the metabolism of neuroactive and vasoactive amines in the central nervous system.

In line with the results obtained here, the involvement of Oct-1 in stress defense has been demonstrated in several other studies^2,31^.

The highest level of the primate-specific Oct-1Z transcript was observed in human brain, mainly in the cortex. Human cerebral cortex is a complex structure (neocortex) which is involved in higher-order brain functions. Understanding the molecular basis of neocortex development rely largely upon the identification of primate and human-specific genes which are active in the neural cells of the developing neocortex^49^. So far, 35 primate-specific genes have been described which are expressed in the human fetal neocortex. Fifteen more genes expressed in the human fetal neocortex were found only in humans^24^. All these genes play an essential role in cortex development. For example, the primate-specific TMEM14B induces the expansion of neocortical surface area^50^, human-specific ARHGAP11B and NOTCH2NL promote basal progenitor proliferation^51,52^, while the primate-specific isoform of PLEKHG6 regulates neurogenesis and neuronal migration^53^.

The DEG analysis has demonstrated that the primate-specific Oct-1Z isoform is directly involved in the processes which are associated with brain development. Oct-1Z is engaged in the development and maturation of central nervous system through the regulation of migration and differentiation of neurons as well as synapse formation and plasticity. In particular, the RELN gene plays a role in neuron layering in the cerebral cortex and cerebellum^54^. Oct-1Z overexpression leads to a sixfold increase in RELN expression in differentiating IMR32 neural cells. The protein encoded by the FABP7 gene is important for the establishment of the radial glial fiber system in the developing brain^55^. The product of the BCAN gene takes part in the brain extracellular matrix development^56^. NOG is required for the growth and patterning of the neural tube^57,58^. NR2E1 is an orphan receptor^59^, COL3A1, which encodes the collagen type III monomer precursor, inhibits neuronal migration and activates the RhoA pathway^60^, NEFL encodes the neurofilament light chain constituting axoskeleton^61^, and NTRK2 is a neurotrophic tyrosine receptor kinase^62^.

Our data also demonstrate that Oct-1Z up-regulates a significant number of genes involved in the different aspects of neural cell differentiation and brain development. It participates in the control of brain development, hippocampus development, neural crest cell development, frontal structures morphogenesis, cortex development, neuropeptide hormone activity, and serotonin receptor signaling pathway. The increase in the Oct-1Z levels leads to the increase in the expression of a number of important genes engaged in the chemical synaptic transmission and neuroactive ligand-receptor interaction.

The expression analysis of the currently known primate-specific genes has demonstrated that many of them are preferentially involved in defense responses and brain development^58^.

The presence of the transcription factor isoforms varying in length and N-terminal sequences contributes to the surprising diversity of functions in transcription regulation^63^. The roles of Oct-1Z in stress protection and differentiation regulation may be interconnected since in differentiation, neural cells experience different types of stress (stress and differentiation are tightly connected). The special role which Oct-1Z plays in primates arises from the fact that the translation of this isoform is increased in many types of stress and mediates the protection response to stress in brain and other tissues.

## Methods

### Cell cultures and human cells transduction

Human Neuroblastoma IMR32 and Namalwa Burkitt lymphoma cell lines (Russian Cell Culture Collection, Institute of Cytology, St. Petersburg, Russia) were used in the study. IMR32 cells were maintained in MEM containing 10% FBS, 100 U/mL penicillin, and 100 µg/mL streptomycin. Namalwa cells were maintained in DMEM containing 10% FCS, 100 U/mL penicillin, and100 µg/mL streptomycin. Vira Power Lentiviral Expression System (Invitrogen) was used to obtain the stable transduction of the IMR32 cell line according to the manufacturer’s protocol. The lentiviral constructs pL-Oct-1Z-3FLAG, pL-Oct-1L-3FLAG (C-end), and pLenti6/V5 (empty vector) were used to obtain the IMR32-Oct-1Z, IMR32-Oct-1L, and IMR32-E.V. lines stably expressing the Oct-1Z-3FLAG and Oct-1L-3FLAG isoforms and the Empty vector, respectively. Blasticidin was used to maintain stably transformed cells and was removed from the media 3 days before experiments. Differentiation of the Neuroblastoma IMR32 cell line was performed in MEM containing 10% FCS, 100 U/mL penicillin, 100 µg/mL streptomycin, and 2.5 µM BrdU. Differentiation was carried out for 16 days, with culturing medium being changed every 3 days.

### Constructs

The pL-Oct-1Z-3FLAG and pL-Oct-1L-3FLAG (C-end) constructs were produced by inserting a copy of the human Oct-1 isoform-coding sequence into the pLenti6/V5 expression vector (Invitrogen).

The pcDNA3.1-Oct-1A-3FLAG, pcDNA3.1-Oct-1L-3FLAG, pcDNA3.1-Oct-1Z-3FLAG, and pcDNA3.1-Oct-1ΔZ -3FLAG(C-end) constructs were produced by inserting a copy of the human Oct-1 isoform-coding sequence into the pcDNA3.1 vector. Oct-1Z cDNA contains both the uORF and the Oct-1Z coding ORF, while in the Oct-1DZ, 285 bp starting from the ATG codon of the L exon were deleted, hence, it contains only the Oct-1Z ORF.

### RNA isolation and qRT-PCR analysis

Human tissue-specific RNA was included in the FirstChoice Human Total RNA Survey Panel (Ambion). Human brain-specific RNA was purchased from Yorkshire Bioscience Ltd (Cat # YBS0126, YBS0110, YBS0127, YBS0105, YBS0121, YBS0116, YBS0125, YBS0124, YBS0112, YBS0117, YBS0113, YBS0120, YBS0114, YBS0107, YBS0129). RNA was isolated from the cell lines using Trizol. Oct-1 mRNA levels were measured by reverse transcription and real-time PCR and normalized to the levels of the glucuronidase B (GUS)-encoding mRNA. Reverse transcription was performed using 2 µg of total RNA and the Maxima First Strand cDNA Synthesis Kit for RT-qPCR (Thermo Scientific) according to the manufacturer’s recommendations. cDNA was synthesized using mixed oligo(dT)_18_ and random hexamer primers. Primers used were as follows: Oct-1A Forw5’-TATTCAAAATGGCGGACGGA-3’; Oct-1L Forw5’-CCACCCCAAACTGCTACCTGT-3’; Oct-1A,L Rev 5’-GTTTCTGACGGATTGTTCATTC-3’; Oct-1Z Forw5’-ATCTATGGAGAGTGGAGATGGCA-3'; Oct-1Z Rev 5’-TGGAAGTGGATCATCACAAAGATC-3'. GUS-s 5’- CGTGGTTGGAGAGCTCATTTGGA -3’ and GUS-as 5’- ATTCCCCAGCACTCTCGTCGGT -3’. Q-PCR was performed in the LightCycler96 thermal cycler (Roche, USA). The standard reaction mixture (25 μl) contained the corresponding primer pairs, cDNA equivalent to 50 ng of total RNA, and qPCRmix-HS SYBR Master Mix (Evrogen). Real-time PCR conditions were as follows: 55 °C for 2 min, 95 °C for 5 min, followed by the 40 cycles of 95 °C for 10 s and 59 °C for 30 s (signal acquisition temperature). Oct-1 mRNA levels following tunicamycin treatment were measured in three independent biological replications. In each case, the measurements were carried out in at least three replicates, and mean values were calculated. The obtained raw data was processed with the aid of the LightCycler96 Instrument software. In the tunicamycin treatment experiments, qRT-PCR data were processed by calculating ∆Ct. To calculate relative Oct-1Z expression in different human brain regions and tissues, data were processed using the ΔΔCt method.

### In vitro transcription and translation of Oct-1 isoforms

For the pcDNA3.1-Oct-1A-3FLAG, pcDNA3.1-Oct-1L-3FLAG, and pcDNA3.1-Oct-1Z-3FLAG constructs, 1 µg of plasmid DNA was used in the transcription and translation reaction using the TNT Coupled Reticulocyte Lysate System (Promega) according to the manufacturer’s instructions. The obtained proteins were separated by SDS–PAGE in 8% polyacrylamide gel and visualized by Western blot analysis using the rabbit anti-Oct-1Z or mouse anti-FLAG antibodies.

### Nuclear extracts preparation

Nuclear extracts were prepared from the Namalwa cells using NE-PER™ Nuclear and Cytoplasmic Extraction Reagents (Thermo Scientific) according to the manufacturer’s recommendations. To prepare nuclear extracts, 2 × 10^6^ cells were taken. To study the expression of the isoforms, nuclear extracts (5 µg per line) were resolved by SDS–PAGE in 8% gel and visualized by Western blot analysis using the rabbit anti-Oct-1Z, rabbit anti-Oct-1L, and rabbit anti-Oct-1(total) antibodies (Abcam).

### Rabbit polyclonal antibodies preparation and purification

To obtain human Oct-1Z-specific antibodies, rabbits were immunized with the Oct-1Z isoform N-end-specific synthetic peptide MKTRMKIFVMIHFHLMNS conjugated to PEGilated KLH using the Imject™ Maleimide-Activated mcKLH Spin Kit (Thermo Scientific™) according to the manufacturer's protocol. The Thermo Scientific Imject Maleimide-Activated mcKLH Spin Kit contains stabilized lyophilized Maleimide-Activated mcKLH (in PBS with stabilizer) and accessory components enabling easy production of the ready-to-use immunogens for subsequent immunization. Rabbits were immunized s.c. with the five doses containing 250 µg of conjugate (peptide-KLH) each. Blends were obtained nine days after the third and the last dose. The obtained antibodies were precipitated with ammonium sulfate and purified from the anti- KLH antibodies using affinity columns. To avoid cross-reactivity, anti-Oct-1Z antibodies were let through the affinity column with the bound Oct-1L and Oct-1A-specific peptides.

### Quantification and verification of anti-Oct1Z antibodies

The Oct1Z protein obtained in the transcription-translation reaction was applied on the 8% SDS gel, Western Blotting was performed, and the nitrocellulose membrane was hybridized with the anti-Oct1Z antibodies at 1:50, 1:100,1:200, 1:400, and 1:1000 dilutions on the Mini Protean II Multi Screen (Bio-Rad). Anti-Rabbit HRP conjugate was used as the secondary antibody at the dilution of 1:5000.

Samples were loaded in triplicate. The Amersham ECL prime kit was used to visualize the signal. ECL signal was quantified using the ChemiDoc MP Imaging System (Bio-Rad). The obtained antibodies specifically recognized the corresponding peptide and the corresponding Oct-1Z isoform in Western blots at 1:200 dilution. The specificity of the anti-Oct-1Z antibody towards its target was verified by three methods: (1) using in vitro transcription and translation of Oct-1A, Oct-1L, and Oct-1Z isoforms and Western blot hybridization with anti-Oct-1Z antibody and anti-FLAG antibody (Fig. 2). (2) using genetically modified HeLa cells with RNAi (Supplementary Fig S1d). HeLa cells stably overexpressing Oct-1∆Z were used, since the corresponding construct allows producing the mRNA enabling maximum Oct-1Z protein translation levels. RNAi was obtained by transfecting HeLa-Oct-1∆Z cells with the short hairpin RNA (shRNA) vectors, either Oct-1-specific or scrambled as a positive control. (3) antibodies were also validated using Western blotting in the presence of the synthetic peptide (100 ng added to the antibody hybridization mixture) (Supplementary Fig S1d).

Experiments on rabbits were carried out in the Institute of Gene Biology, Russian Academy of Sciences. Authors confirm that all experiments on rabbits were carried out and all methods were used in accordance with the relevant guidelines and regulations. Authors confirm that all experiments involving animals were approved by the Ethics Committee of the Institute of Gene Biology, Russian Academy of Sciences. This study was carried out in compliance with the ARRIVE guidelines.

### Antibodies

The following antibodies were also used: Rabbit anti-Oct-1L, Rabbit anti-Oct-1(total) antibody (Abcam, Ab 66132), Mouse anti-FLAG antibody (Sigma, F9291), Rabbit anti-beta-actin (AbCam, ab 6276) and Rabbit anti-Lamin B1 (AbCam, Ab65986), Goat anti-mouse HRP (Jackson ImmunoResearch, 115-035-174) and Goat anti-Rabbit HRP (Jackson ImmunoResearch, 111-035-144), and Rabbit anti-Oct-1L^3^. For the more detailed description of antibodies, see Supplementary Table S5.

### Western-blot analysis

Protein extracts (10 μg) or nuclear protein extracts (2 μg) mixed with the Laemmli loading buffer containing DTT were incubated at 40 °C for 10 min, then applied on the 8% SDS-PAGE, and transferred onto the nitrocellulose membrane (GE Healthcare) in 25 mM Tris, 192 mM glycine, and 20% methanol, with subsequent membrane blocking with 5% nonfat milk in PBS for 1 h at room temperature. Antibodies against FLAG (Oct-1-FLAG), against total Oct-1, Oct-1L, or Oct-1Z isoforms, Lamin B, or beta-actin were used for the Western blot assay (Table S5). 10 µg of cell protein extract were loaded into each lane. Lamin B or beta-actin was used as a loading control for normalization. Membranes were probed with the primary antibodies in PBS supplemented with 0.1% Tween-20 (PBS-T) or with the serum obtained from the rabbits immunized with the Oct-1Z peptide in 5% nonfat milk in PBS-T at 4 °C overnight, and then washed 3 times for 20 min with PBS-T and incubated for 1 h at RT with the anti-mouse HRP antibodies (Santa-Cruz Biotechnology, sc-2005, 1:5,000) or anti-rabbit HRP antibodies (Santa-Cruz Biotechnology, sc-2005, 1:5.000). After four additional washings steps with PBS-T, signal detection was performed according to the standard protocol using the ECL- reagent (GE Helthcare). The results of the Western blot assay were vizualized, and the signal was quantitatively assessed using the ChemiDoc MP Imaging System (Bio-Rad) with the aid of the Bio-Rad Image Lab software. In each experiment, the measurements were made in at least triplicates, and mean values were calculated.

### Cell stress

Namalwa cells were cultured in the complete DMEM medium with 0.5 µg/mL of camptothecin for 24 h. Neuroblastoma IMR32-EmptyVector, IMR32-Oct-1Z, and IMR32-Oct-1L cells were cultured in the complete MEM medium. ER stress was caused by culturing cells in the presence of 1 µg/mL of tunicamycin for 8 h and Camptothecin-induced stress, in the presence of 0.5 µg/mL of camptothecin, for 24 h. Culturing under hypoxic conditions was preformed in the atmosphere containing 0.5% O_2_ for 24 h. Culturing under oxidative stress conditions was performed in the complete MEM medium in the presence of 100 µM or 200 µM H_2_O_2_ for 24 h. Culturing under low glucose conditions was performed in the complete MEM medium containing 0.5% glucose.

Cell viability under oxidative stress was measured using the CellTiter 96 AQ One Solution Assay. The percentage of living cells relative to untreated cells is presented. Cells were treated with H_2_O_2_ in the concentration of 100 µM at 37 °C for 24 h.

### Constructs

#### Transfection and dual luciferas assay

To study the role of the Oct-1Z isoform in the development of cellular response to stress, the pGL4-Firefly Luciferase Reporter Vectors with Cellular Stress Response Elements– pGL4.42[luc2P/HRE/Hygro] and pGL4.39[luc2P/ATF6 RE/Hygro] (Promega) were used. IMR32-Empty Vector and IMR32-Oct-1Z cell lines were transfected with the above-mentioned plasmids together with the pRL-Tk plasmid (Promega) using the TransFast reagent according to the manufacturer’s instructions. After transfection, cells were cultured in the complete MEM medium at 37 °C with 5% CO_2_ for 24 h. After the end of the culturing period, cells were subjected to hypoxia or ER stress for 24 h as described above. Then, Dual Luciferase assay was carried out using the Dual-Glo Luciferase Kit (Promega). Luciferase signal was detected using the GloMax96 device with the 0.5 s integration time.

### Sequencing library preparation

Human16-day differentiating neuroblastoma IMR32-EmptyVector and neuroblastoma IMR32-Oct-1Z cells were obtained in triplicate and mRNA was isolated using the NEBNext Poly(A) mRNA Magnetic Isolation Module (NEB#E7490). NEBNext Ultra II Directional RNA Library Prep Kit for Illumina (NEB #E7760) was used with 3 ug of total RNA for sequencing library construction. RNA libraries were prepared using the standard Illumina protocols. Double-stranded cDNA, ligation reaction products, and PCR reaction products were purified using the AgencourtAMPure XP beads.

### Illumina sequencing and bioinformatics pre-processing

Molar library concentrations were measured using the KAPA Library Quant qPCR kit (Kapa Biosystems), and library size distribution was assessed after PCR (12 cycles) using Agilent BioAnalyzer. Human mRNA profiles of the 16-day differentiating neuroblastoma IMR32-Empty Vector and neuroblastoma IMR32 cells with the overexpression of the primate-specific Oct-1Z transcription factor isoform were generated by deep sequencing, in triplicate, using Illumina NovaSeq. FASTQ files were obtained using the bcl2fastq v2.20 Conversion Software (Illumina). Recording format for the quality data strings—Phred 33. Reads were mapped to the human genome (hg38) using the hisat software. In each library, about 89–90% of the obtained data on average was uniquely aligned. The htseq-count package was used to calculate the number of reads which were mapped to known genes (ncbi—entrezID). The obtained values (cpm—countpermillion) for each gene for each library were combined into a single matrix for further analysis. Filtration, normalization by the TMM method, variance estimation, and assessment of differentially expressed genes were performed using the edgeR module. Genes in which cpm was lower that 1 in any of the three libraries were considered to be low-expressed. GeneID: Gene name (gene symbol) obtained from the database—org.Hs.eg.db based on the entrezID. LogFC: Logarithm to the base 2 of foldchange. FDR: p-value with multiple testing correction. Genes with positive LogFC values were considered as upregulated and with the negative LogFC values, as downregulated. After filtering out low-expressing genes, 15,108 entries remained.

### Functional enrichment analysis of DEGs

Gene Ontology screening was performed (GO; DAVID (david.abcc.ncifcrf.gov/home.jsp): GOTERM BP FAT (biological process), GOTERM CC FAT (cellular component), and KEGG Pathway^64^ (www.genome.jp/kegg/pathway.html)). DAVID calculates the modified Fisher’s Exact P-value to reveal GO or molecular pathway enrichment. The P-value of < 0.01 was chosen as a cut-off criterion.

### Statistics

Statistical analysis was performed using the GraphPad software (GraphPad Software, San Diego, CA, USA). The unpaired Student’s t-test was used to generate P-values. Error bars represent SEM (*P < 0.05, and **P < 0.01).

### Accession numbers

Oct-1Z mRNA—GenBank MT294127. RNA-Seq data—GEO GSE153980.

## Supplementary Information

Supplementary Information.
