# Supplementary material for: Primate-specific stress-induced transcription factor POU2F1Z protects human neuronal cells from stress
Source: Sci Rep. 2021 Sep 22;11:18808. doi: 10.1038/s41598-021-98323-y (PMC8458439; doi:10.1038/s41598-021-98323-y)
Supplement: Supplementary file 1 — Supplementary Information. [file 41598_2021_98323_MOESM1_ESM.pdf]

Supplementary Materials

Primate-specific stress-induced transcription factor POU2F1Z protects human neuronal cells from stress

Alexander G. Stepchenko, Tatiana N. Portseva, Ivan A. Glukhov, Alina P. Kotnova, Bella M. Lyanova, Sofia G. Georgieva, Elizaveta V. Pankratova

Supplementary Figure S1

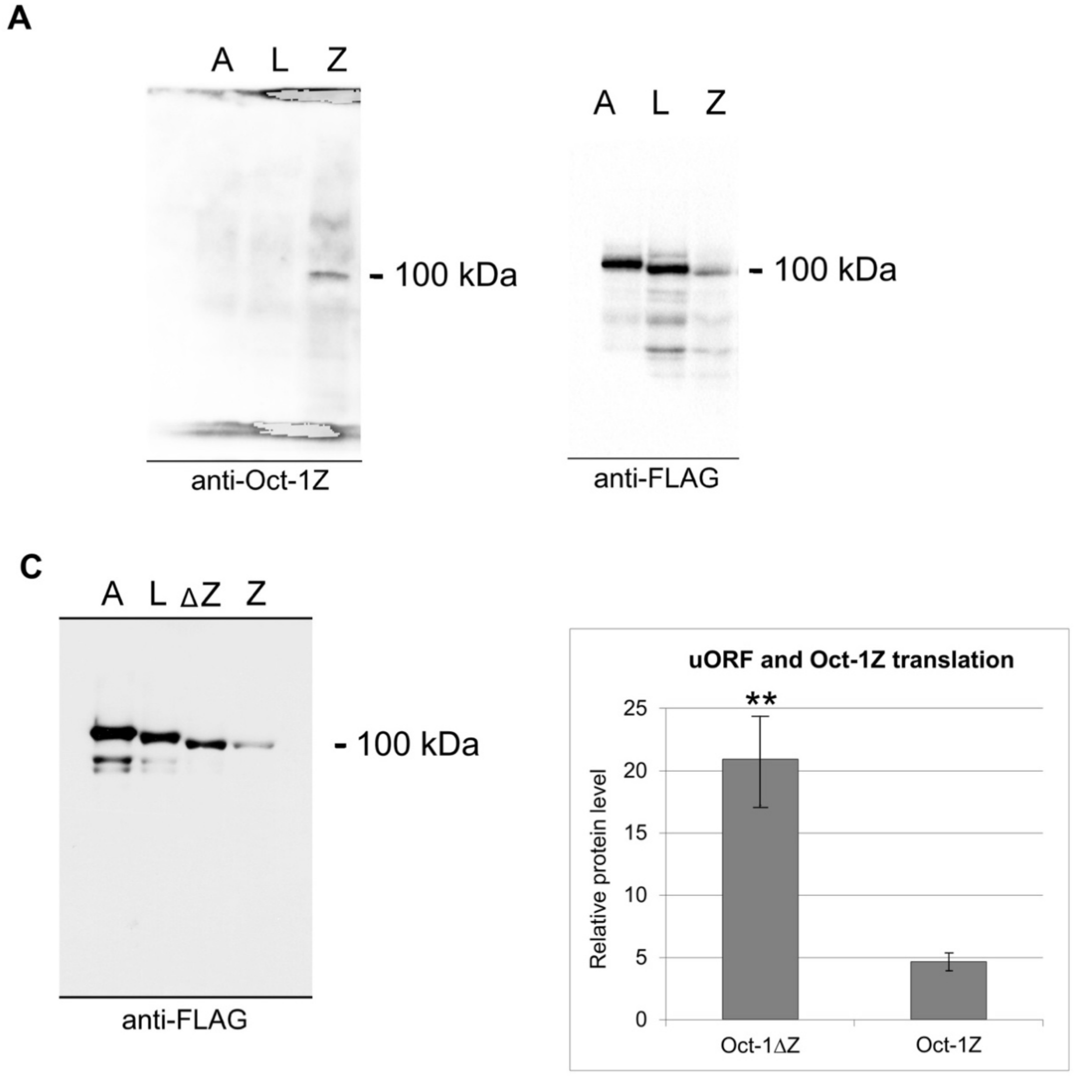

Supplementary figure S1. A, C - The complete cropped spot images shown in Fig. 3 are shown. Original, unprocessed images are represented. Before hybridization, the upper border of the blot (A - anti-FLAG) was cut from the top due to a defect in the gel. Where possible, images with a gray background are shown. Weak borders are indicated by a black line. **C** - Deletion of 285 bp at the 5'-end enhances the translation of Oct-1Z. Error bars indicate S.E.M. for six replicates. t-tests have been performed to compare the means (**P < 0.01).

d

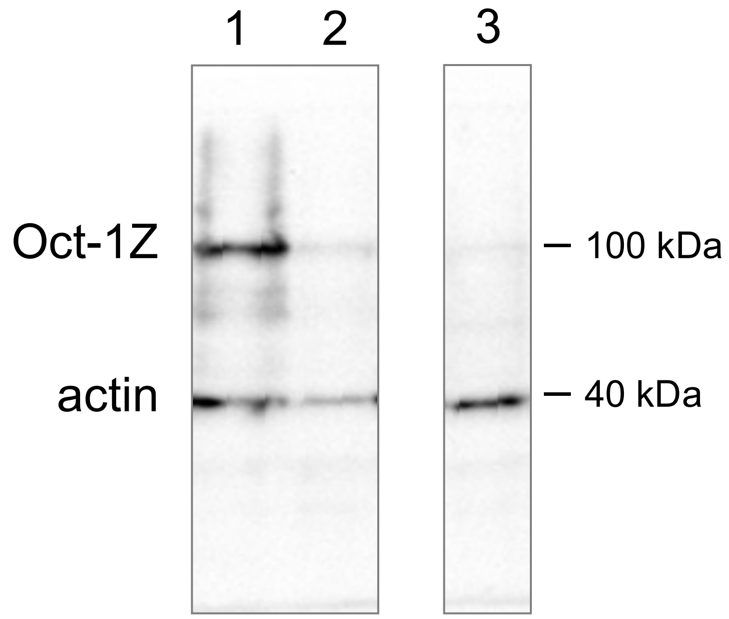

Western blotting results for the rabbit anti-Oct-1Z polyclonal antibody validation using HeLa cells overexpressing Oct-1ΔZ isoforms (HeLa-Oct-1ΔZ). Whole cell extracts (15 μg) were applied on the 8% SDS gel. After WB, the membrane with transferred proteins was probed with the anti Oct-1Z antibody (1:200 dilution). (Line 1) HeLa –Oct-1ΔZ+scrambled siRNA (CAAAAATTCTCCGAACGTG) whole cell lysates; (line 2) Oct-1Z knockdown in HeLa –Oct-1ΔZ after transfection with Oct-1-targeting siRNA (GCCAAGACCUUCAAACAAA). As it is in lane 1, Oct-1Z knockdown is shown along with the scrambled RNA as a control; (lane 3) WB membrane was probed with the anti Oct-1Z antibody (1:200 dilution) in the presence of the target peptide (MKTRMKIFVMIHFHLMNS) (100 ng). Blocking peptide prevented antibody binding with the Oct-Z protein. Actin was used as a loading control.

The obtained antibodies were used exclusively for Western blot and were not tested for IP, IF, and ChIP. The results were reproducible in all experimental replicates. The results of the anti-Oct1Z antibody testing allow to conclude that this antibody is specific, selective and reproducible in the context of our experiments.

Supplementary Figure S2

a1

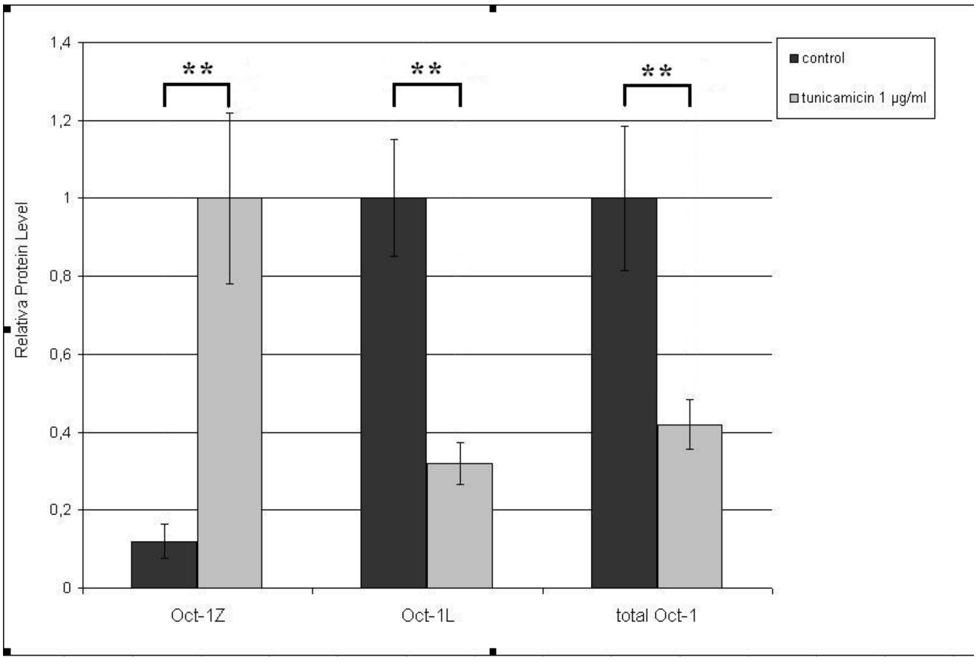

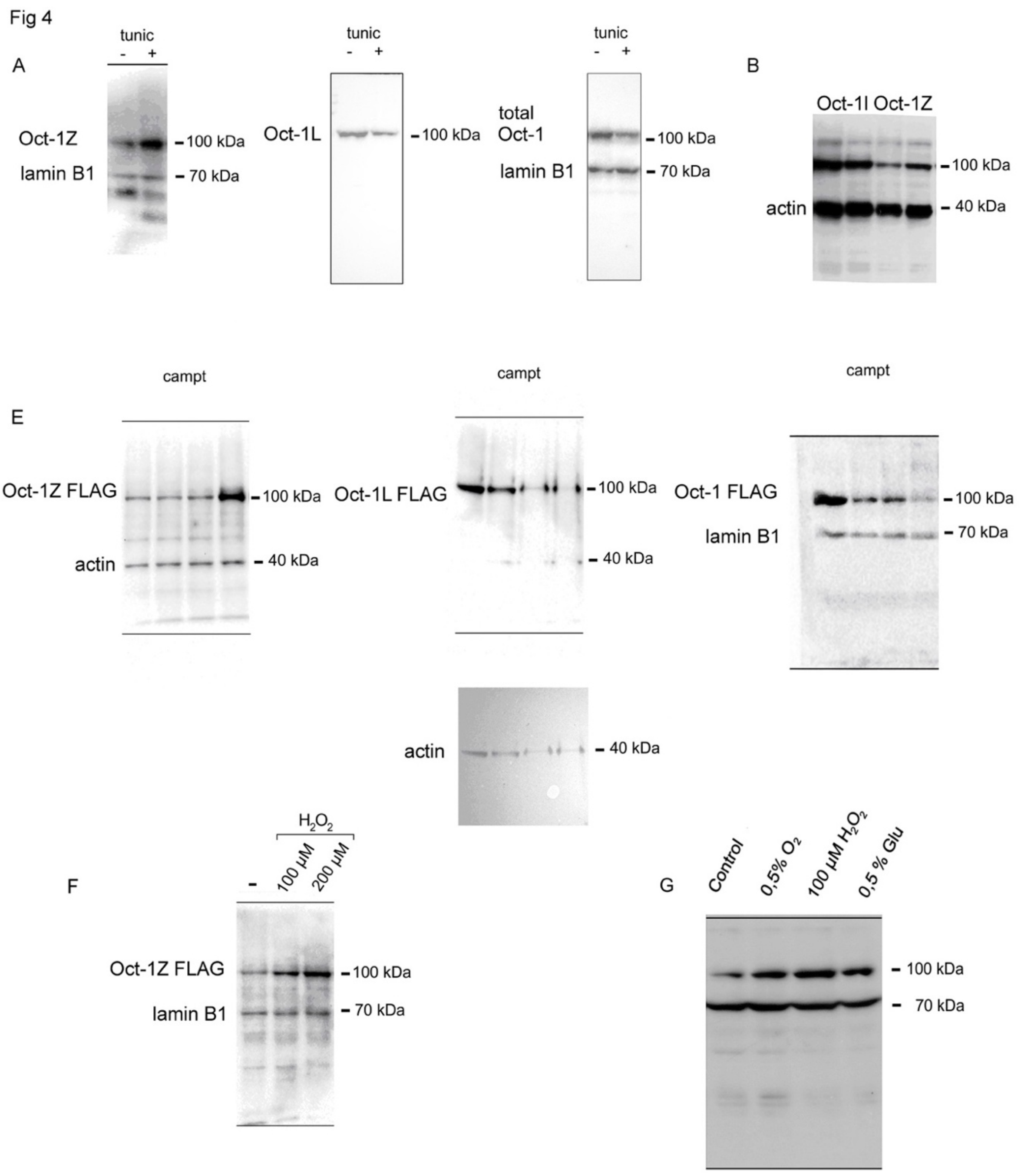

Supplementary Figure S2. A(1) Changes in Oct-1Z, Oct-1L, and total Oct-1 relative levels in the Namalwa cells treated with tunicamycin (1 μg/ml, 24 h) based on three independent experiments. The levels of lamin B have been used as normalizator. The graphs show mean ± S.E.M. for three independent experiments. t-tests have been performed to compare the means (**$P < 0.01$).

A, B, E, F- full cropped images of spots shown in Fig. 4. Original, unprocessed images are presented. Before hybridization, the upper border of blot A (Oct-1Z) and blot B (anti-FLAG) were cut off due to a defect in the gel, and the upper border of blot E (actin) was cut in order to remove the signal from the Oct-1 protein. Where possible, images with a gray background are shown. Weak borders are indicated by a black line.

Supplementary Figure S3. Changes in the morphology of IMR32 cells during differentiation. Arrows indicate axons and dendrites.

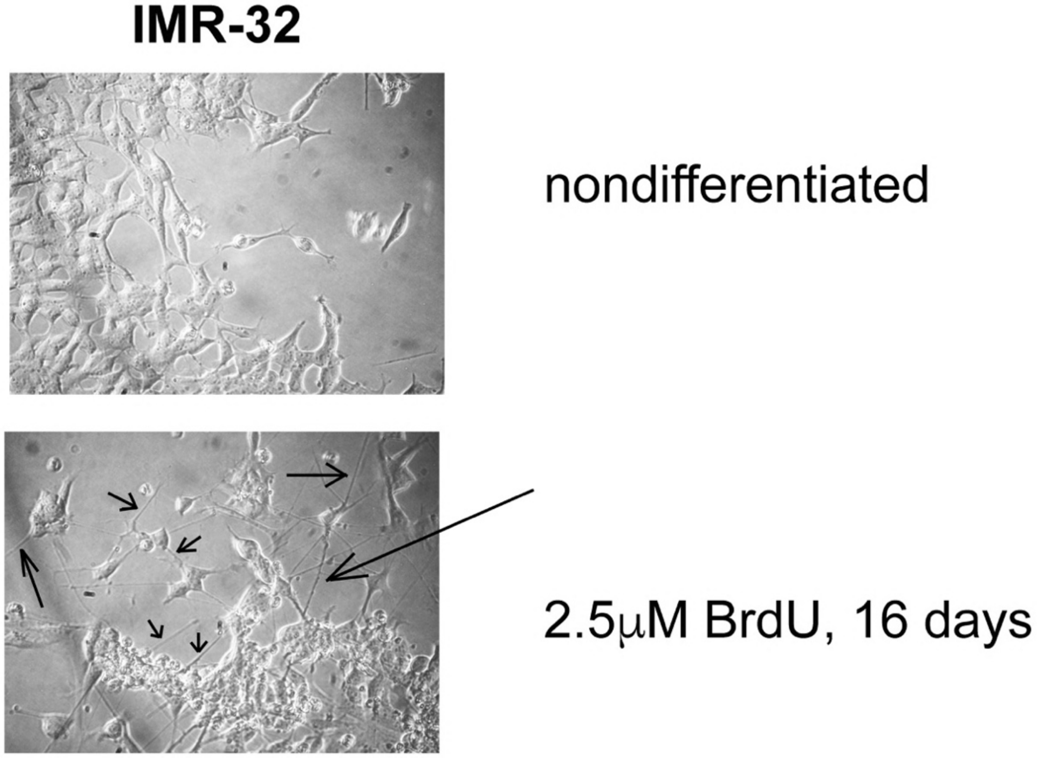

Table S1. Fold change for DEGs related to cell stress reponse.

Gene		logFC	FDR
BMP2	Bone Morphogenetic Protein 2	0,790465	0,0030194
CYP1A1	Cytochrome P450 Family 1 Subfamily A Member 1	1,592439	0,0004273
EGLN3	Egl-9 Family Hypoxia Inducible Factor 3	0,845513	0,0008767
EPAS1	Endothelial PAS Domain Protein 1	0,851714	0,0087809
AJUBA	Ajuba LIM Protein	1,950392	1,53E-05
MMP2	Matrix Metallopeptidase 2	0,942373	0,0002448
PRKCB	Protein Kinase C Beta	1,172733	0,0064174
FOXO1	Forkhead Box O1	0,798376	0,0021585
LOXL2	Lysyl Oxidase Like 2	1,225044	0,0022562
MICAL2	Microtubule Associated Monooxygenase, Calponin And LIM Domain Containing 2	1,625042	0,0080592
MOXD1	Monooxygenase DBH Like 1	2,177091	0,0010798
HTR1D	5-Hydroxytryptamine Receptor 1D	2,811714	0,0008054
DHRS2	Dehydrogenase/Reductase 2	1,392586	0,0001936
EPHX1	Epoxide Hydrolase 1	0,484992	0,0073544
INMT	Indolethylamine N-Methyltransferase	2,508797	0,0034662
MAOB	Monoamine Oxidase B	1,578949	0,0011361
TLR2	Toll Like Receptor 2	2,553062	0,0001831
PMAIP1	Phorbol-12-Myristate-13-Acetate-Induced Protein 1	0,642607	0,0010261

LogFC: Logarithm of the fold change to the base 2
FDR: p-value with multiple testing correction

Table S2. Fold change of DEGs related to brain development.

GeneID	Gene Name	logFC	FDR
RELN	Reelin	2,58208788	0,00093413
FABP7	Fatty Acid Binding Protein 7	1,18689007	0,00668849
MECOM	MDS1 And EVI1 Complex Locus	1,74319142	0,00013875
BCAN	Brevican	1,76470041	0,00635196
DLX1	Distal-Less Homeobox 1	1,24276364	0,0029048
NOG	Noggin	1,40812548	0,00207153
NR2E1	Nuclear Receptor Subfamily 2 Group E Member 1	2,92744332	0,00155435
COL3A1	Collagen Type III Alpha 1 Chain	1,87336543	0,00060587
NEFL	Neurofilament Light	0,70499751	0,00491973
NTRK2	Neurotrophic Receptor Tyrosine Kinase 2	0,67360226	0,00063504
NCOA1	Nuclear Receptor Coactivator 1	0,47774673	0,0097895
PTPRC	Protein Tyrosine Phosphatase Receptor Type C	1,19926828	0,00608371
TACC1	Transforming Acidic Coiled-Coil Containing Protein 1	0,75037582	0,0010887
TBX3	T-Box Transcription Factor 3	0,71211662	0,00039978

LogFC: Logarithm of the fold change to the base 2
FDR: p-value with multiple testing correction

Table S3. Fold change of DEGs related to chemical synaptic transmission and neuroactive ligand-receptor interaction.

GeneID	Gene Name	logFC	FDR
HTR1D	5-Hydroxytryptamine Receptor 1D	2,8117143	0,0008054
HTR2B	5-Hydroxytryptamine Receptor 2B	1,9745457	0,0094166
HTR6	5-Hydroxytryptamine Receptor 6	0,8994167	0,0039564
CARTPT	CART Prepropeptide	1,1481407	0,0041766
SLITRK5	SLIT And NTRK Like Family Member 5	1,4306596	0,0006591
CACNA1E	Calcium Voltage-Gated Channel Subunit Alpha1 S	1,3843007	0,0031397
DOC2A	Double C2 Domain Alpha	1,1156924	0,0020410
GRIA3	Glutamate ionotropic receptor AMPA type subunit 3	0,5346372	0,0129984
GRIN2A	Glutamate ionotropic receptor NMDA type subunit 2A(GRIN2A)	1,9165916	0,0001882
GRIN2B	Glutamate Ionotropic Receptor NMDA Type Subunit 2B	2,0040879	0,0105245
HAP1	Huntingtin Associated Protein 1	0,9050802	0,0134611
LRFN1	Leucine Rich Repeat And Fibronectin Type III Domain Containing 1	1,1059045	0,0105245
PDE7B	Phosphodiesterase 7B	0,9397073	0,0050098
KCNC4	Potassium Voltage-Gated Channel Subfamily C Member 4	0,6269311	0,0037497
PENK	Proenkephalin	2,6264241	7,45E-06
PLP1	Proteolipid Protein 1	1,1586830	0,0025269
SLC12A4	Solute Carrier Family 12 Member 4	1,0992621	0,0067777
SLC6A4	Solute Carrier Family 6 Member 4	2,2407266	0,0022562
SSTR1	Somatostatin Receptor 1	3,7229911	8,24E-05
SST	Somatostatin	2,6648534	8,12E-05
SYPL1	Synaptophysin Like 1	0,6808999	0,002466
TACR1	Tachykinin Receptor 1	1,2778147	0,0088071
CHRNA9	Cholinergic Receptor Nicotinic Alpha 9 Subunit	1,5144314	0,0011100
CHRNA6	Cholinergic Receptor Nicotinic Alpha 6 Subunit	1,3288835	0,0068195
GABRA1	Gamma-aminobutyric acid typeA receptor alpha1 subunit	1,0428977	0,0025823
GABRA5	Gamma-aminobutyric acid type A receptor alpha5	-0,627618	0,0002577
GABRE	Gamma-aminobutyric acid type A receptor epsilon subunit	1,4042341	0,0055226
GABRP	Gamma-aminobutyric acid type A receptor pi subunit	1,923614	0,0002017

LogFC: Logarithm of the fold change to the base 2

FDR: p-value with multiple testing correction

Table S4. Fold change of DEGs related to cell-matrix adhesion.

GeneID	Gene Name	logFC	FDR
CD96	CD96 Molecule	1,23330063	0,00628346
COL3A1	Collagen Type III Alpha 1 Chain	1,87336543	0,00060587
ECM2	Extracellular Matrix Protein 2	1,71248122	0,00046559
FERMT2	Fermitin Family Member 2	0,57409058	0,0012601
FBLN5	Fibulin 5	1,85800358	0,00170477
ITGA11	Integrin Subunit Alpha 11	1,30313485	0,00067875
ITGA3	Integrin Subunit Alpha 3	1,00361139	0,00129298
ITGA6	Integrin Subunit Alpha 6	1,4066804	0,00069223
ITGA8	Integrin Subunit Alpha 8	2,06427021	7,45E-06
ITGB3	Integrin Subunit Beta 3	1,87758516	0,00342105
ITGB5	Integrin Subunit Beta 5	1,82102954	8,12E-05
NPNT	Nephronectin	1,68868926	0,00053655
VCAM1	Vascular Cell Adhesion Molecule 1	1,91079506	0,00035508
VCL	Vinculin	1,24188852	0,00013238

LogFC: Logarithm of the fold change to the base 2
FDR: p-value with multiple testing correction

Table S5.

Antibody	Manufacturer	Cat. Number	RRID
Rabbit anti Oct1<br>WB dilution 1:500	AbCam	Ab 66132	**RRID:AB_1141895**
Mouse anti FLAG BioM2<br>WB dilution 1:1000	Sigma	F9291	**RRID:AB_439698**
Rabbit anti Lamin B1<br>WB dilution 1:2000	AbCam	Ab65986	**RRID:AB_1140888**
Goat anti mouse HRP<br>WB dilution 1:5000	Jackson ImmunoResearch	115-035-174	**RRID:AB_2338512**
Goat anti Rabbit HRP<br>WB dilution 1:5000	Jackson ImmunoResearch	111-035-144	**RRID:AB_2307391**
Mouse anti-beta actin antibody<br>WB dilution 1:5000	AbCam	ab 6276	**RRID 2223210**
